# Lysine pathway metabolites and the risk of type 2 diabetes and cardiovascular disease in the PREDIMED study: results from two case-cohort studies

**DOI:** 10.1186/s12933-019-0958-2

**Published:** 2019-11-13

**Authors:** Cristina Razquin, Miguel Ruiz-Canela, Clary B. Clish, Jun Li, Estefania Toledo, Courtney Dennis, Liming Liang, Albert Salas-Huetos, Kerry A. Pierce, Marta Guasch-Ferré, Dolores Corella, Emilio Ros, Ramon Estruch, Enrique Gómez-Gracia, Montse Fitó, Jose Lapetra, Dora Romaguera, Angel Alonso-Gómez, Lluis Serra-Majem, Jordi Salas-Salvadó, Frank B. Hu, Miguel A. Martínez-González

**Affiliations:** 1 Department of Preventive Medicine and Public Health, University of Navarra, Pamplona, Spain; 2 IdiSNA, Navarra Institute for Health Research, Pamplona, Spain; 3 CIBER Fisiopatología de la Obesidad y Nutrición (CIBERObn), Instituto de Salud Carlos III, Madrid, Spain; 4 Broad Institute of MIT and Harvard University, Cambridge, USA; 5 Department of Nutrition, Harvard T.H. Chan School of Public Health, Boston, Spain; 6 Department of Epidemiology, Harvard T.H. Chan School of Public Health, Boston, USA; 7 Department of Biostatistics, Harvard T.H. Chan School of Public Health, Boston, USA; 8 Human Nutrition Unit, Faculty of Medicine and Health Sciences, Institut d’Investigació Sanitària Pere Virgili, Rovira i Virgili University, Reus, Spain; 9 Department of Preventive Medicine, University of Valencia, Valencia, Spain; 10 Lipid Clinic, Department of Endocrinology and Nutrition, Institut d’Investigacions Biomediques August Pi Sunyer (IDI- BAPS), Hospital Clinic, University of Barcelona, Barcelona, Spain; 11 Department of Internal Medicine, Institut d’Investigacions Biomediques August Pi Sunyer (IDI-BAPS), Barcelona, Spain; 12 Department of Preventive Medicine, University of Malaga, Malaga, Spain; 13 Cardiovascular and Nutrition Research Group, Institut de Recerca Hospital del Mar (IMIM), Barcelona, Spain; 14 Department of Family Medicine, Research Unit, Distrito Sanitario Atención Primaria Sevilla, Seville, Spain; 15 Instituto de Investigación Sanitaria de Palma (IdISPa), University Hospital of Son Espases, Palma de Mallorca, Spain; 16 Department of Cardiology, University Hospital of Alava, Vitoria, Spain; 17 Research Institute of Biomedical and Health Sciences, University of Las Palmas de Gran Canaria, Las Palmas, Spain; 18 Channing Division for Network Medicine, Department of Medicine, Brigham and Women’s Hospital and Harvard Medical School, Boston, USA

**Keywords:** Biomarkers, Metabolites, Cardiovascular disease, Type 2 diabetes, Dietary intervention

## Abstract

**Background:**

The pandemic of cardiovascular disease (CVD) and type 2 diabetes (T2D) requires the identification of new predictor biomarkers. Biomarkers potentially modifiable with lifestyle changes deserve a special interest. Our aims were to analyze: (a) The associations of lysine, 2-aminoadipic acid (2-AAA) or pipecolic acid with the risk of T2D or CVD in the PREDIMED trial; (b) the effect of the dietary intervention on 1-year changes in these metabolites, and (c) whether the Mediterranean diet (MedDiet) interventions can modify the effects of these metabolites on CVD or T2D risk.

**Methods:**

Two unstratified case-cohort studies nested within the PREDIMED trial were used. For CVD analyses, we selected 696 non-cases and 221 incident CVD cases; for T2D, we included 610 non-cases and 243 type 2 diabetes incident cases. Metabolites were quantified using liquid chromatography–tandem mass spectrometry, at baseline and after 1-year of intervention.

**Results:**

In weighted Cox regression models, we found that baseline lysine (HR_+1 SD increase_ = 1.26; 95% CI 1.06–1.51) and 2-AAA (HR_+1 SD increase_ = 1.28; 95% CI 1.05–1.55) were both associated with a higher risk of T2D, but not with CVD. A significant interaction (p = 0.032) between baseline lysine and T2D on the risk of CVD was observed: subjects with prevalent T2D and high levels of lysine exhibited the highest risk of CVD. The intervention with MedDiet did not have a significant effect on 1-year changes of the metabolites.

**Conclusions:**

Our results provide an independent prospective replication of the association of 2-AAA with future risk of T2D. We show an association of lysine with subsequent CVD risk, which is apparently diabetes-dependent. No evidence of effects of MedDiet intervention on lysine, 2-AAA or pipecolic acid changes was found.

*Trial registration* ISRCTN35739639; registration date: 05/10/2005; recruitment start date 01/10/2003

## Background

Due to unhealthy diet and lifestyles in the context of ageing populations, the global epidemic of cardiovascular disease (CVD) and type 2 diabetes (T2D) has reached unexpected dimensions. In order to stop this pandemic, it is of utmost importance to find robust biomarkers for both early detection and risk prediction of CVD [1] and T2D [2].

Previous studies have shown that circulating 2-aminoadipic acid (2-AAA) levels were associated with obesity and metabolic syndrome [3, 4] and had the ability to predict the risk of future T2D [5]. The metabolite 2-AAA results from the degradation pathway of lysine (Additional file 1: Fig. S1), an essential amino acid. However, lysine has not been consistently associated with metabolic syndrome or T2D in previous studies [3]. In the pathway of lysine degradation (Additional file 1: Fig. S1) there is also another metabolite, pipecolic acid, that may be implicated in other diseases such as human diabetic corneal stroma [6]. Thus, it seemed important to further explore lysine degradation pathway in relation to T2D to find potential biomarkers and/or underlying biological mechanisms.

On the other hand, T2D and CVD share many common etiologic factors, and as explained above, 2-AAA has been postulated as a predictor factor for T2D [5]. Thus, it seems important to study the potential role of 2-AAA in the development of CVD. In the same context, lysine and 2-AAA have also been implicated in the development of other CVD risk factors, such as obesity and metabolic syndrome [3]. Consequently, the role of metabolites involved in lysine degradation (trough 2-AAA) pathway should also be explored in association with CVD and whether the presence of T2D may modify this association should be studied.

One study suggested that 2-AAA may be modified by diet because a study reported significantly higher levels of 2-AAA after a beef meal compared to a baked herring meal [7], proposing a potential dietary effect on the regulation of the pathway.

In the frame of the PREDIMED study, a large nutritional intervention trial with Mediterranean diet (MedDiet) for the primary prevention of CVD, we assessed the following hypotheses: (a) baseline (or 1-year changes) in plasma levels of lysine, 2-AAA or pipecolic acid are associated with higher incidence of T2D and CVD; (b) the intervention with MedDiet induced changes in lysine, 2-AAA or pipecolic acid after 1-year; and (c) an intervention with MedDiet is able to attenuate the potential adverse effects of lysine, 2-AAA or pipecolic acid on T2D or CVD risk.

## Methods

**Fig. 1:**
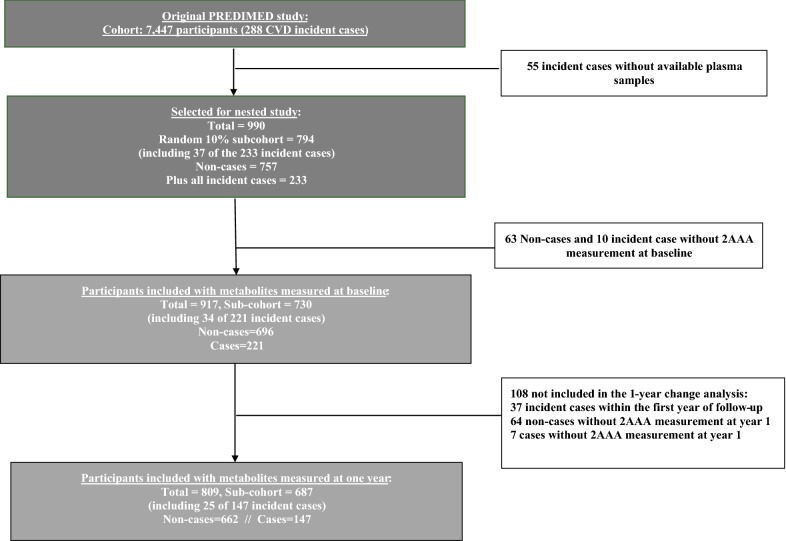
Flow-chart of the case-cohort design for CVD. *CVD* cardiovascular disease

**Fig. 2:**
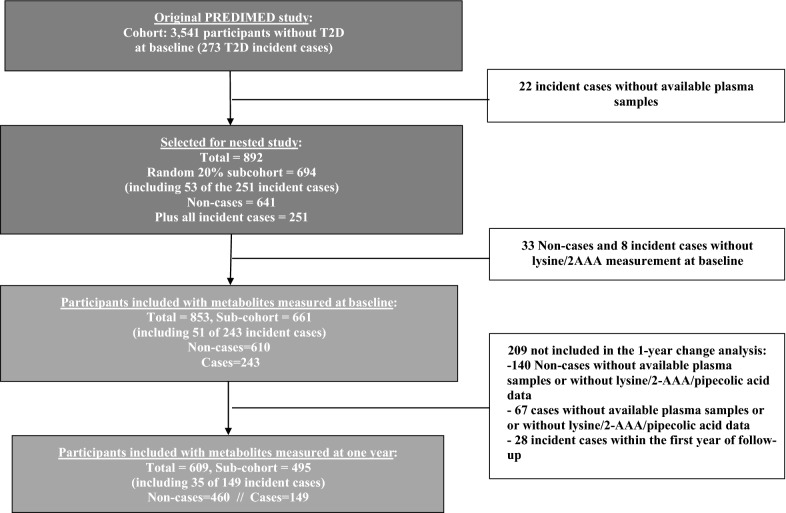
Flow-chart of the case-cohort design for T2D. *T2D* type 2 diabetes

To analyze the association of lysine, 2-AAA or pipecolic acid with CVD or T2D, we used two sets of case-cohort studies (Figs. 1, 2) nested within the PREDIMED trial (Trial registration: ISRCTN35739639; registration date: 05/10/2005; recruitment start date 01/10/2003; http://www.predimed.es). PREDIMED was a primary cardiovascular prevention trial testing Mediterranean diets, as described elsewhere [8, 9]. Briefly, 7447 participants (men aged 55–80 years and women aged 60–80 years), initially free of CVD but at high cardiovascular risk, were allocated to 3 dietary interventions: (1) a MedDiet supplemented with extra-virgin olive oil (MedDiet + EVOO); (2) a MedDiet supplemented with mixed nuts (MedDiet + nuts); or (3) a control diet (low-fat diet).

In the first nested case-cohort study (Fig. 1), cases were all primary CVD events with available blood samples and the subcohort was a random sample of all the trial. We included a random sample of ~ 10% of PREDIMED participants at baseline (the subcohort) and 233 incident cases of CVD with available blood samples which occurred during a median follow-up of 4.8 years (55 of the 288 incident cases of the PREDIMED trial had no available plasma samples). We also excluded 73 participants (63 non-cases and 10 cases) because of unavailable 2-AAA or lysine metabolites data (Fig. 1). Finally, 917 participants were included in our analysis: 221 incident cases and 730 participants in the subcohort (including 34 overlapping cases). In addition, 809 participants (687 participants in the subcohort and 147 cases, including 25 overlapping cases) had available plasma samples after 1-year of follow-up and were included in the analyses of metabolite changes at 1 year (Fig. 1).

In the design of the second case-cohort study (Fig. 2) we considered that 3541 participants did not have T2D at baseline in the full PREDIMED cohort and we only considered them for the second case-cohort design, as they were candidates to develop new-onset T2D. Among them, there were 273 incident cases of T2D observed during follow-up. The present study comprised a random selection of 694 participants (approximately 20%) from the roster of all eligible subjects of the PREDIMED (subcohort), together with all incident cases of T2D with available plasma sample that occurred during a median follow-up of 3.8 years of intervention (cases). Baseline measurements for the metabolites were available for 610 non-cases and 243 incident cases (51 of the cases were overlapped with the subcohort). On the other hand, 669 participants, 516 non-cases and 153 (35 of them overlapped with the subcohort) cases that occurred after 1 year of follow-up, had available 1-year follow-up samples. Among them, 609 subjects had 1-year lysine, 2-AAA and pipecolic acid measurements and were finally included in the 1-year change analyses (Fig. 2).

The Research Ethics Committees for each of the recruitment centres approved the study protocol and all participants provided written informed consent.

### Ascertainment of CVD and T2D cases

The primary endpoint of the PREDIMED trial was a composite outcome of non-fatal acute myocardial infarction, non-fatal stroke and cardiovascular death. Physicians, blinded with respect to the intervention, reviewed yearly, in each recruitment center, all the participants’ medical charts to assess any incident CVD outcome. Other sources of information (also blinded with respect to the intervention), such as consultation of the National Death Index, were used to ascertain incident cases. Then, anonymized information was sent from each recruitment center to a blinded central Event Ascertainment Committee who finally adjudicated the events.

The PREDIMED protocol included T2D as a pre-specified secondary endpoint of the trial among participants initially free of diabetes. At baseline, prevalent T2D was identified by clinical diagnosis and/or use of antidiabetic medication. The diagnosis of incident T2D during follow-up has been described elsewhere [9, 10] and followed the American Diabetes Association criteria [11]. Blinded study physicians collected information on the outcomes in the yearly ad hoc reviews of the participants’ medical charts. Also, information on incident cases of T2D was collected from continuous contact with participants and primary health care physicians, and annual follow-up visits. Blinded to the intervention assignment, the Clinical End-Point Committee adjudicated the events according to standard criteria.

### Covariate assessment

At baseline and at yearly follow-up visits, participants completed a questionnaire collecting lifestyle information, educational achievement, history of illnesses, medication use, and family history of disease. Physical activity was assessed using the validated Spanish version of the Minnesota Leisure-Time Physical Activity questionnaire [12].

### Study samples and metabolite profiling

Fasting blood samples were collected at baseline and after 1 year of follow-up. After centrifugation, plasma EDTA was collected, and aliquots were coded and kept refrigerated until they were stored at − 80 °C. Pairs of samples (baseline and first-year visits from each participant) were randomly ordered and shipped on dry ice to the Broad Institute for the metabolomics analyses.

Liquid chromatography tandem mass spectrometry (LC–MS) was used to measure polar plasma metabolites. Negative ion mode, targeted MS analyses of 2-AAA were conducted as described previously [5]. Briefly, LC–MS samples were prepared from plasma (30 µL) via protein precipitation with the addition of four volumes of 80% methanol containing inosine-15N4, thymine-d4 and glycocholate-d4 internal standards (Cambridge Isotope Laboratories; Andover, MA). The samples were centrifuged (10 min, 9000×*g*, 4 °C) and the supernatants were analyzed using an ACQUITY UPLC (Waters, Milford MA) coupled to a 5500 QTRAP triple quadrupole mass spectrometer (AB SCIEX, Framingham, MA). Extracts (10 µL) were injected directly onto a 150 × 2.0 mm Luna NH2 column (Phenomenex; Torrance, CA). The column was eluted at a flow rate of 400 µL/min with initial conditions of 10% mobile phase A (20 mM ammonium acetate and 20 mM ammonium hydroxide in water) and 90% mobile phase B (10 mM ammonium hydroxide in 75:25 v/v acetonitrile/methanol) followed by a 10 min linear gradient to 100% mobile phase. MS data were acquired using multiple reaction monitoring scans tuned using authentic reference standards. The ion spray voltage was − 4.5 kV and the source temperature was 500 °C. Raw data were processed using MultiQuant 2.1 software (SCIEX, Framingham MA). High resolution, positive ion mode analyses of lysine and pipecolic acid were conducted using a hydrophilic interaction liquid chromatography (HILIC) LC–MS method as described previously [13]. Briefly, data were acquired using a Nexera X2 U-HPLC system (Shimadzu Scientific Instruments; Marlborough, MA) coupled to a Q Exactive orbitrap mass spectrometer (Thermo Fisher Scientific; Waltham, MA). Metabolites were extracted from plasma (10 µL) using 90 µL of 74.9:24.9:0.2 v/v/v acetonitrile/methanol/formic acid containing stable isotope-labeled internal standards (valine-d8, Isotec; and phenylalanine-d8, Cambridge Isotope Laboratories; Andover, MA). The extracts were centrifuged (10 min, 9000×*g*, 4 °C), and the supernatants were injected onto a 150 × 2 mm Atlantis HILIC column (Waters; Milford, MA). The column was eluted isocratically at a flow rate of 250 µL/min with 5% mobile phase A (10 mM ammonium formate and 0.1% formic acid in water) for 1 min followed by a linear gradient to 40% mobile phase B (acetonitrile with 0.1% formic acid) over 10 min. Polar metabolite MS analyses were carried out using electrospray ionization in the positive ion mode using full scan analysis over m/z 70–800 at 70,000 resolution and 3 Hz data acquisition rate. Additional MS settings were: ion spray voltage, 3.5 kV; capillary temperature, 350 °C; probe heater temperature, 300 °C; sheath gas, 40; auxiliary gas, 15; and S-lens RF level 40. Raw data were processed using Progenesis QI software (NonLinear Dynamics) for feature alignment, nontargeted signal detection, and signal integration. Compound identities were confirmed using reference standards and reference samples. Internal standard peak areas were monitored for quality control and to ensure system performance throughout analyses. Pooled plasma reference samples were analyzed at intervals of approximately 20 samples as an additional quality control and to determine analytical reproducibility [14]. Coefficients of variation (CV) for 2-AAA were 42.4% and 41.5% in the CVD (n = 126 pooled samples) and T2D (n = 124 pooled samples) datasets, respectively. Lysine CVs were 6.8% and 1.9% and pipecolic acid CVs were 10.6% and 2.3% in the CVD (n = 100 pooled samples) and T2D (n = 92 pooled samples) datasets, respectively.

### Statistical analysis

The analyses in both case-cohort studies were run in parallel and following the same scheme, so we will describe the common methods. Only small differences concerning adjustments for multivariable models were introduced and will be summarized below.

#### Baseline analyses

Baseline lysine, 2-AAA and pipecolic acid values were normalized and scaled in multiples of 1 SD with Blom’s inverse normal transformation [15]. We fitted weighted Cox regression models using Barlow weights to account for the over-representation of cases, as recommended for case-cohort designs [16]. We calculated hazard ratios (HR) and their 95% confidence intervals (95% CI) for CVD or T2D by quartiles of baseline lysine, 2-AAA or pipecolic acid. Quartile cut-off points were generated based on the distributions of each metabolite among subcohort. We conducted tests of linear trend by examining an ordinal score based on the median value in each quartile of lysine, 2-AAA or pipecolic acid in the multivariable models. Follow-up time was calculated from the date of enrollment to the date of diagnosis of CVD/T2D for cases, and to the date of the last visit or the end of the follow-up period for non-cases (December 1, 2010). Progressively further adjusted models were designed: first, a model adjusted for age, sex and intervention group was run; secondly, we included a multivariable adjustment including as covariates BMI (kg/m^2^), smoking (never/current/former), leisure-time physical activity (metabolic equivalent task [MET]s-min/day), educational level (primary vs secondary or higher), hypertension and dyslipidemia for the association with T2D, and, also including baseline T2D and family history of CVD for the association with CVD. An additional model was designed for the association with T2D including also the adjustment for plasma baseline glucose (both continuous and quadratic term included in the model).

In order to assess the potential relationship of this pathway and other factors underlying the development of T2D, the correlations (Pearson) between 2-AAA and baseline glucose, insulin, LDL-c and HDL-c were analyzed.

As an additional analysis, we wanted to observe the potential modifying role of prevalent T2D on the associations between baseline lysine, 2-AAA or pipecolic acid with CVD. A new variable combining the baseline diabetic status and the levels of each metabolite (below/above the median) was introduced into the models. Moreover, two independent models for diabetics and for non-diabetics were fitted.

To assess if the intervention with MedDiet was an effect modifier of the associations between baseline values of the metabolites and the risk of T2D or CVD, fully adjusted models for the case-cohorts of both CVD and T2D were fitted and multiplicative independent product-terms were used to assess the potential interactions. Potential multiplicative interactions between the intervention group (MedDiet + EVOO, MedDiet + nuts or control) and the dichotomous variable for lysine, 2-AAA and pipecolic acid (defined by the values below/above its respective median) were tested with the likelihood ratio test. Moreover, three new independent variables combining the intervention groups (MedDiet + EVOO, MedDiet + nuts, or control) and the dichotomous variable defined for each metabolite by its median (below or above) were created and introduced separately in fully adjusted models to analyze the effects of each metabolite stratified by intervention group. The reference category was the group of subjects allocated to the control group of the trial and the group of participants below the median of each metabolite. Additionally, adjustment for propensity scores that used 30 baseline variables to estimate the probability of assignment to each of the intervention groups and robust variance estimators were used to take into account that a small percentage of participants were non-individually randomized to the intervention groups and minor imbalances in baseline covariates existed in the trial [9].

#### One-year changes

To assess the associations between 1-year changes in lysine, 2-AAA and pipecolic acid and subsequent risk of CVD or T2D, only the cases that occurred after 1-year follow-up were used.

First of all, we calculated the changes between 1-year measurements and baseline for each metabolite and then normalized the differences using Blom’s inverse normal transformation. We used the same Cox regression models that for baseline analyses but including as independent variable 1-year changes for each metabolite and adjusting for their respective baseline values.

We also assessed the combined effects of intervention and 1-year changes for the three metabolites and tested the interactions between intervention groups and 1-year changes. As we did at baseline, propensity scores adjustment and robust variance estimators were used in these models.

To analyze the effects of intervention on metabolites changes, we only included participants in the selected subcohorts representing a random sample of the full roster of the trial (CVD and T2D cases not included in subcohorts were excluded). We used a linear regression model with the intervention as the main independent variable and 1-year changes in lysine, 2-AAA and pipecolic acid (residual change obtained after a regression of the 1-year metabolite value on baseline value), respectively, as dependent variables and adjusted for the following independent covariates: age, sex, BMI, smoking, leisure-time physical activity, hypertension and dyslipidemia. Again, propensity scores adjustment and robust variance were used in these models.

## Results

Baseline characteristics of participants, included in T2D and CVD case-cohort designs, are shown in Tables 1 and 2 respectively. In the baseline comparison of characteristics between incident T2D cases and non-cases, we found that baseline glucose levels among future diabetes cases pointed to a pre-diabetic baseline stage. In fact, according to the American Diabetes Association criteria for prediabetes [17] considering only fasting glucose, the 47% of the subjects included in the subcohort appeared to present baseline prediabetes whereas the 86% of T2D incident cases presented prediabetes at baseline. On the other hand, subsequent incident CVD cases (as compared to non-cases) were at baseline older and more likely to be diabetic, sedentary and smokers.

**Table 1:** Baseline characteristics of the participants according to the case-cohort design for T2D

	Subcohort (n = 661)^a^	T2D cases (n = 243)
Age (years)	66.5 (5.7)	66.4 (5.7)
BMI (kg/m^2^)	29.8 (3.6)	30.8 (3.4)
LTPA (METs-min/day)	240 (241)	248 (233)
Total energy intake (kcal/day)	2279 (560)	2335 (620)
Adherence to MedDiet (14 items)	8.65 (1.91)	8.52 (1.82)
Sex, female (%)	61.7	54.7
Family history of CVD (%)	30.1	22.2
Education, secondary or higher (%)	25.3	23.5
Plasma baseline glucose (mg/dl)	100 (15)	117 (18)
Dyslipidaemia (%)	84.9	79.4
Hypertension (%)	90.8	95.9
Smoking		
Never (%)	60.2	53.1
Current (%)	16.8	25.1
Former (%)	23	21.8
Intervention group		
Control (%)	32.1	35.4
MedDiet + EVOO (%)	30.4	30.5
MedDiet + nuts (%)	37.5	34.2

*BMI* body mass index, *LTPA* leisure time physical activity, *MedDiet* Mediterranean diet, *CVD* cardiovascular disease, *EVOO* extra-virgin olive oil

^a^Including 51 overlapping cases

**Table 2:** Baseline characteristics of the participants according to the case-cohort design for CVD

	Subcohort (n = 730)^a^	CVD cases (n = 221)
Age (years)	67 (5.8)	69.5 (6.5)
BMI (kg/m^2^)	29.8 (3.6)	29.6 (3.8)
LTPA (METs-min/day)	261 (258)	232 (235)
Total energy intake (kcal/day)	2330 (615)	2379 (687)
Adherence to MedDiet (14 items)	8.8 (1.93)	8.4 (1.81)
Sex, female (%)	56.9	39.8
Family history of CVD (%)	25.9	18.6
Diabetes prevalence (%)	47	65.2
Education, secondary or higher (%)	25	19
Dyslipidaemia (%)	74.4	57.9
Hypertension (%)	83.3	82.8
Smoking		
Never (%)	62.6	44.8
Current (%)	12.5	19.5
Former (%)	24.9	35.7
Intervention group		
Control (%)	29.6	36.7
MedDiet + EVOO (%)	37.2	34.4
MedDiet + nuts (%)	33.2	29

*BMI* body mass index, *LTPA* leisure time physical activity, *MedDiet* Mediterranean diet, *CVD* cardiovascular disease, *EVOO* extra-virgin olive oil

^a^Including 34 overlapping cases

We present in parallel the results obtained for the T2D case-cohort and CVD case-cohort studies. In Table 3 we show the association of baseline lysine, 2-AAA or pipecolic acid with CVD or T2D. We found that higher levels of lysine, 2-AAA and pipecolic acid were associated with T2D but the strongest association was found for 2-AAA, which was significantly associated with an increased risk of T2D in the basic model adjusted for sex, age and intervention group (HR_+1 SD increase_ = 1.29; 95% CI 1.10–1.52 Table 3), in the multivariable model (HR_+1 SD increase_ = 1.27; 95% CI 1.07–1.50, data not shown) and even in the plasma glucose-adjusted model (HR_+1 SD increase_ = 1.28; 95% CI 1.05–1.55; Table 3). This association was monotonic across increasing quartiles of 2-AAA (p for trend = 0.026). Similarly, we observed that higher levels of lysine were also associated with a higher risk of T2D in the fully adjusted model (HR_+1 SD increase_ = 1.26; 95% CI 1.06–1.51; Table 3). When the three metabolites were simultaneously included in the fully adjusted model the effects were attenuated but they were still evident for 2-AAA and lysine (HR_+1 SD increase 2-AAA_ = 1.21; 95% CI 0.99–1.47 and HR_+1 SD increase lysine_ = 1.20; 95% CI 0.99–1.44; Table 3). To deepen into the role of the pathway in the development of T2D, we analyzed the potential correlations between baseline 2-AAA and glucose, insulin, HDL-c and LDL-c for the T2D case-cohort. These correlations, though statistically significant, were not particularly high. The correlation between baseline 2-AAA and fasting glucose was r = 0.161; p < 0.001 (n = 904) and the correlation between baseline 2-AAA with insulin was r = 0.179; p < 0.001 (n = 713). In contrast, an inverse correlation was found between 2-AAA and HDL-c (r = − 0.134; p < 0.001; n = 757) and between 2-AAA and LDL-c (r = − 0.116; p = 0.016; n = 742).

**Table 3:** Association [HR (95% CI)] between baseline levels of lysine, 2-aminoadipic acid and pipecolic acid with cardiovascular disease or type 2 diabetes

	Q1	Q2	Q3	Q4	p linear trend	1 SD increase
Baseline lysine						
Outcome: T2D						
Basic model^a^	Ref.	1.10 (0.70–1.71)	1.47 (0.95–2.28)	1.24 (0.80–1.92)	0.189	1.11 (0.96–1.29)
MV adjusted model^b^	Ref.	1.19 (0.67–2.11)	1.56 (0.88–2.75)	1.66 (0.96–2.86)	0.038	1.26 (1.06–1.51)
MV adjusted for 2-AAA and pipec.	Ref.	1.17 (0.67–2.07)	1.52 (0.85–2.70)	1.50 (0.87–2.60)	0.113	1.20 (0.99–1.44)
Outcome: CVD						
Basic model^a^	Ref.	0.86 (0.55–1.34)	0.96 (0.61–1.51)	0.94 (0.60–1.47)	0.897	0.99 (0.81–1.20)
MV adjusted model^c^	Ref.	0.80 (0.48–1.29)	1.01 (0.62–1.65)	0.84 (0.51–1.38)	0.718	0.98 (0.82–1.17)
MV adjusted for 2-AAA and pipec.	Ref.	0.78 (0.49–1.35)	0.99 (0.61–1.60)	0.82 (0.49–1.35)	0.636	0.98 (0.82–1.17)
Baseline 2-AAA						
Outcome: T2D						
Basic model^a^	Ref.	0.95 (0.59–1.54)	1.40 (0.89–2.21)	1.92 (1.23–1.54)	0.001	1.29 (1.10–1.52)
MV adjusted model^b^	Ref.	1.28 (0.66–2.50)	1.51 (0.82–2.77)	1.94 (1.03–3.63)	0.026	1.28 (1.05–1.55)
MV adjusted for lysine and pipec.	Ref.	1.32 (0.68–2.59)	1.43 (0.77–2.63)	1.81 (0.97–3.37)	0.050	1.21 (0.99–1.47)
Outcome: CVD						
Basic model^a^	Ref.	1.11 (0.70–1.74)	0.94 (0.59–1.51)	1.21 (0.76–1.90)	0.532	1.06 (0.90–1.23)
MV adjusted model^c^	Ref.	1.18 (0.73–1.91)	0.84 (0.51–1.40)	1.13 (0.69–1.83)	0.894	1.00 (0.85–1.18)
MV adjusted for lysine and pipec.	Ref.	1.20 (0.74–1.95)	0.86 (0.51–1.43)	1.19 (0.71–1.99)	0.766	1.01 (0.85–1.20)
Baseline pipecolic acid						
Outcome: T2D						
Basic model^a^	Ref.	1.32 (0.86–2.02)	1.13 (0.72–1.78)	1.46 (0.93–2.31)	0.171	1.13 (0.96–1.32)
MV adjusted model^b^	Ref.	1.24 (0.72–2.14)	0.97 (0.55–1.71)	1.37 (0.78–2.41)	0.411	1.13 (0.92–1.13)
MV adjusted for lysine and 2-AAA	Ref.	1.07 (0.62–1.87)	0.86 (0.49–1.51)	1.32 (0.74–2.36)	0.506	1.08 (0.88–1.32)
Outcome: CVD						
Basic model^a^	Ref.	0.69 (0.44–1.09)	0.77 (0.49–1.21)	0.93 (0.61–1.42)	0.948	1.03 (0.88–1.20)
MV adjusted model^c^	Ref.	0.71 (0.45–1.11)	0.68 (0.42–1.11)	0.84 (0.55–1.38)	0.610	0.99 (0.83–1.18)
MV adjusted for lysine and 2-AAA	Ref.	0.70 (0.43–1.14)	0.70 (0.43–1.14)	0.88 (0.55–1.41)	0.669	0.99 (0.82–1.20)

*CVD* cardiovascular disease, *T2D* type 2 diabetes, *MV* mutivariable

^a^Adjusted for age, sex and intervention

^b^Basic model additionally adjusted for BMI, smoking, dyslipidemia, hypertension and baseline plasma glucose

^c^Basic model additionally adjusted for BMI, smoking, dyslipidemia, hypertension, T2D, family history of CVD

In the main analyses of the association of lysine, 2-AAA and pipecolic acid with CVD we did not observe any significant result (Table 3). We additionally repeated these analyses considering the combined variable prevalent T2D and levels of each metabolite (Additional file 1: Fig. S2), and we found the highest risk of CVD for subjects with both prevalent T2D at baseline and high levels of lysine at baseline. A significant interaction between lysine levels (below or above the median) and T2D at baseline (p = 0.032; Additional file 1: Fig. S2) was observed.

**Fig. 3:**
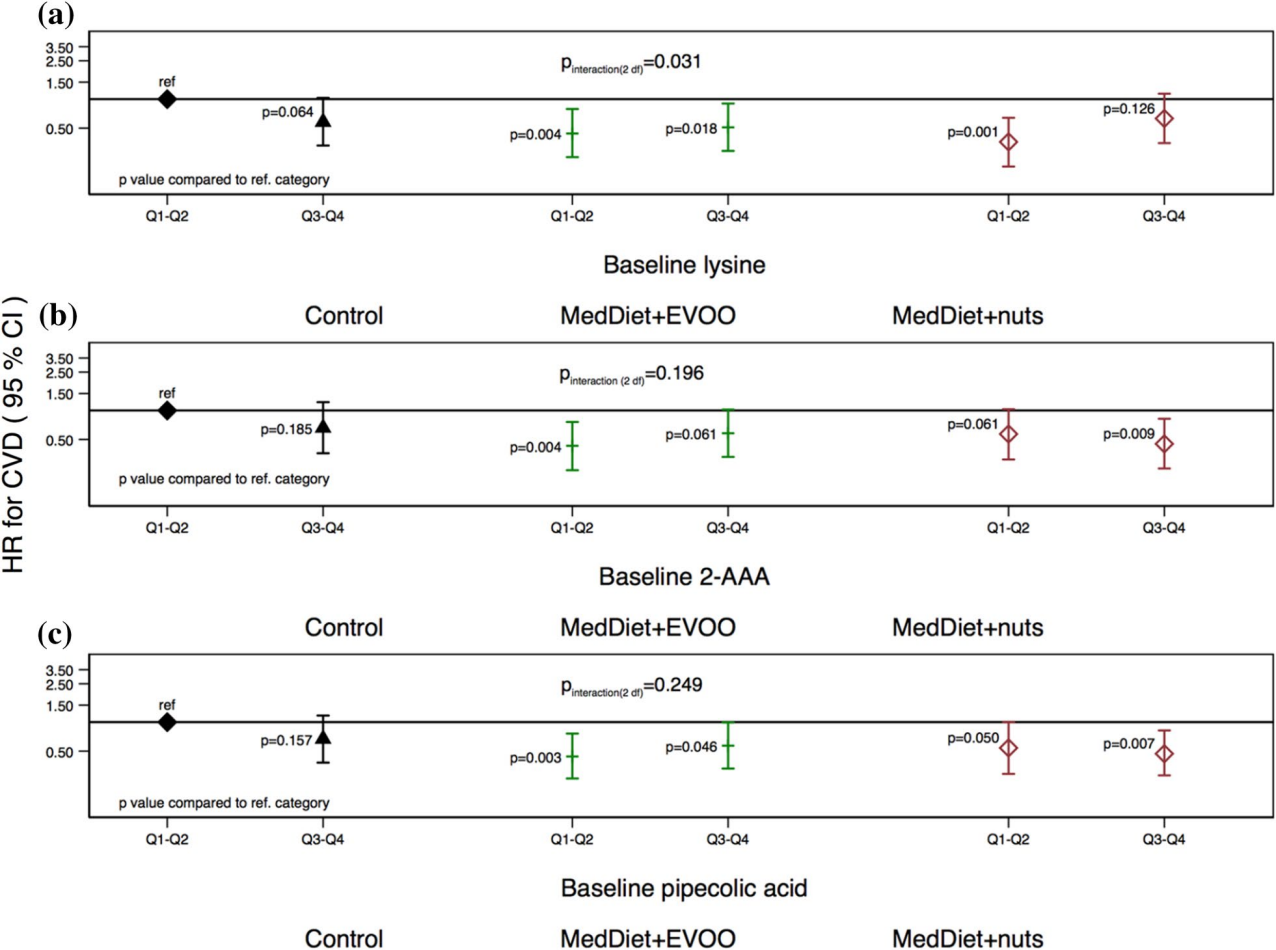
Multivariate adjusted hazard ratios (HRs; 95% confidence intervals [CIs]) of incident cardiovascular disease for quartiles (Q) of baseline **a** lysine, **b** 2-AAA and **c** pipecolic acid stratified by intervention group. p values are referred to the reference category composed of control group subjects with each baseline metabolite below the median

As a next step, we assessed if the dietary intervention with MedDiet was able to modify the effects of baseline levels of lysine, 2-AAA and pipecolic acid on T2D and CVD risk. We did not find any significant interaction for lysine, 2-AAA or pipecolic acid and the risk of T2D (Additional file 1: Fig. S3). The modification effect of the intervention was evident for CVD risk: we found a significant multiplicative interaction between baseline levels of lysine and the intervention group (p interaction-2df = 0.031; Fig. 3), suggesting that subjects with low baseline levels of lysine and allocated to one of the MedDiet intervention groups may present the lowest risk for CVD. Both baseline 2-AAA and pipecolic acid showed the same pattern of association for MedDiet + EVOO group but the interaction was not statistically significant for any of them.

Finally, we analyzed if 1-year changes in lysine, 2-AAA and pipecolic acid were prospectively associated with T2D or CVD risk (Table 4). We found that 1-year increases in lysine, 2-AAA and pipecolic acid were not significantly associated with higher T2D risk (HR_+1 SD increase in lysine_ = 0.80; 95% CI 0.58 to 1.10; HR_+1 SD increase in 2-AAA_ = 1.29; 95% CI 0.98 to 1.70; HR_+1 SD increase in pipecolic acid_ = 1.18; 95% CI 0.88 to 1.58). One-year increases in lysine, 2-AAA or pipecolic acid were neither associated with the risk of CVD after 1-year of intervention (HR_+1 SD increase in lysine_ = 0.99; 95% CI 0.80 to 1.24; HR_+1 SD increase in 2-AAA_ = 0.81; 95% CI 0.63 to 1.04; HR_+1 SD increase in pipecolic acid_ = 1.10; 95% CI 0.87 to 1.37) (Table 4). When the potential effect modification by the intervention was considered (Additional file 1: Fig. S4), we found no significant multiplicative interaction for T2D. However, we found that MedDiet + EVOO appeared to be associated with a reduction in the risk of T2D especially among subjects who increased their levels of lysine (p = 0.004) and pipecolic acid (p = 0.041) after 1-year of intervention compared to the reference group (subjects of the control group with 1-year changes below the median of each metabolite). For CVD, we did not find any pattern of association when stratifying by intervention group (Additional file 1: Fig. S5).

**Table 4:** Association [HR (95% CI)] between 1-year changes of lysine, 2-aminoadipic acid and pipecolic acid with type 2 diabetes and cardiovascular disease

	Q1	Q2	Q3	Q4	p linear trend	1 SD increase	1 SD increase (adjusted for 1-year changes in 2-AAA and pipecolic acid)
1-year change lysine							
Outcome: T2D							
MV adjusted model^b^	Ref.	0.72 (0.37–1.40)	0.43 (0.17–1.07)	0.62 (0.29–1.34)	0.215	0.88 (0.65–1.20)	0.80 (0.58–1.10)
Outcome: CVD							
MV adjusted model^a^	Ref.	1.07 (0.63–1.82)	0.93 (0.52–1.66)	0.93 (0.52–1.64)	0.796	0.95 (0.77–1.18)	0.99 (0.80–1.24)

	Q1	Q2	Q3	Q4	p linear trend	1 SD increase	1 SD increase (adjusted for 1-year changes in lysine and pipecolic acid)
1-year change 2-AAA							
Outcome: T2D							
MV adjusted model^b^	Ref.	1.30 (0.63–2.67)	0.95(0.38–2.36)	1.66 (0.82–3.39)	0.214	1.23 (0–96–1.58)	1.29 (0.98–1.70)
Outcome: CVD							
MV adjusted model^a^	Ref.	0.94 (0.52–1.69)	0.97 (0.55–1.72)	0.66 (0.35–1.21)	0.210	0.84 (0.67–1.05)	0.81 (0.63–1.04)

	Q1	Q2	Q3	Q4	p linear trend	1 SD increase	1 SD increase (adjusted for 1-year changes in lysine and 2-AAA)
1-year change pipecolic acid							
Outcome: T2D							
MV adjusted model^b^	Ref.	1.45 (0.54–3.84)	1.22 (0.43–3.46)	1.42 (0.54–3.71)	0.483	1.08 (0.81–1.44)	1.18 (0.88–1.58)
Outcome: CVD							
MV adjusted model^a^	Ref.	0.49 (0.21–1.12)	0.52 (0.24–1.13)	0.93 (0.47–1.84)	0.593	1.03 (0.83–1.28)	1.10 (0.87–1.37)

*CVD* cardiovascular disease, *T2D* type 2 diabetes, *MV* multivariable, *2-AAA* 2-aminoadipic acid

^a^Multivariable model adjusted for age, sex, intervention, BMI, smoking, dyslipidemia, hypertension, baseline plasma glucose and the respective baseline metabolite

^b^Multivariable model adjusted for age, sex, intervention, BMI, smoking, dyslipidemia, hypertension, T2D, family history of CVD and the respective baseline metabolite

Finally, we found that neither the intervention with MedDiet + EVOO nor with MedDiet + nuts were associated with statistically significant 1-year changes in lysine, 2-AAA or pipecolic acid (Additional file 1: Table S1).

## Discussion

In the frame of the PREDIMED trial we found that (1) baseline lysine and especially 2-AAA circulating levels were associated with future risk of T2D but not with CVD risk; (2) a baseline diabetic status appeared to modify the association between baseline lysine levels and subsequent CVD risk so that only subjects who were diabetic at baseline exhibited an association between higher lysine levels and increased risk of future CVD; (3) 1-year changes in lysine, 2-AAA and pipecolic acid were not associated with subsequent CVD or T2D risk; (4) the MedDiet did not show any significant effect on 1-year changes neither in 2-AAA nor in lysine; (5) the MedDiet supplemented with nuts or with EVOO may be reducing CVD or T2D risk through the regulation of different biological mechanisms than those related to lysine degradation. However, MedDiet may be modifying the risk conferred by high levels of metabolites included in the lysine degradation pathway.

A previous work published by Wang et al. [5], found in two independent cohorts—Framingham Heart Study (FHS)-Offspring Cohort and Malmö Diet and Cancer Study (MDC)-Cardiovascular Cohort-that high 2-AAA predicted the risk of developing T2D in normoglycemic individuals. Our results confirmed this association among subjects at high cardiovascular risk showing that the higher the baseline levels of 2-AAA, the higher the subsequent T2D risk in this Mediterranean population. This is an important replication and update of the results considering that all PREDIMED participants randomly selected for the T2D case-cohort were at high risk of developing CVD and had baseline glucose levels that can be considered as a proxy of a pre-diabetic status. Our findings suggest that it seems possible to predict the risk of developing diabetes conferred by 2-AAA even at a pre-diabetic stage. We found similar results for the association of lysine with T2D but this association was not as robust as that for 2-AAA. A recent small cross sectional study reported that 2-AAA was also associated with obesity and metabolic syndrome in addition to T2D [3]. Consistent with our results, they also reported that lysine presented similar but weaker associations with these metabolic conditions. A mechanism potentially explaining these associations may be that 2-AAA is elevated in response to high glucose levels, leading to increased insulin secretion and promoting mechanisms involved in maintaining glucose homeostasis in early stages of insulin resistance [5]. In the case of lysine, it may be elevated at an early stage of this pathophysiological process to provide the 2-AAA substrate for a likely compensatory mechanism [3]. In concordance with these explanations, lysine was also found to be a predictor of insulin resistance and gestational diabetes during pregnancy in a previous study [18]. Moreover, both 2-AAA and pipecolic have been found to be elevated in diabetic corneas suggesting that this pathway may be also related to diabetes-induced diseases [6].

On the other hand, we observed no association between baseline lysine, 2-AAA or pipecolic acid levels and subsequent incidence of CVD in non-diabetic subjects. However, the association of lysine with CVD was present in subjects with an initial diabetic status. Therefore, it is likely that this association may only be apparent among subjects with preexistent T2D. In this context, diabetic status may behave as an effect modifier of the association between lysine and CVD. The p value for the interaction test was statistically significant, supporting this interpretation. This finding represents a novelty and deserves future replication in further independent cohorts. It can be speculated that this effect modification by pre-existent diabetes may suggest that lysine can be implicated in the development of complications associated with T2D, because high levels of lysine may represent a biomarker of a poorer control of glycemic levels in T2D. This interpretation should be taken with caution and further confirmation is needed.

In addition to the 2-AAA pathway of lysine degradation, L-homoarginine may be also synthesized from lysine catabolism and, this alternative pathway, involving arginine and nitric oxide synthesis [19, 20], may be related to CVD and T2D, as we have previously reported [21, 22].

Regarding the effect of the dietary intervention, we observed no effect of MedDiet on lysine, 2-AAA or pipecolic acid 1-year changes. Additionally, we did not find any significant multiplicative interaction between the MedDiet intervention and 1 year changes in lysine, 2-AAA or pipecolic acid. However, we found that MedDiet may be modifying the effects of baseline lysine on CVD risk as we found a significant interaction between lysine and the intervention. In the same direction, results on 2-AAA and pipecolic acid pointed at the same effect of MedDiet + EVOO (non significant interaction). These results may be suggesting that although there is no a direct effect of diet on the levels of the metabolites, it is possible that MedDiet may be modifying the effects of metabolites on CVD or T2D risk by affecting other mechanisms that may involve different metabolic pathways, inflammation or oxidation. Further studies are needed to clarify the effect of diet on lysine, 2-AAA and pipecolic acid levels and the potential effect modification by dietary interventions on the CVD/T2D risk associated with these metabolites.

Our study presents some limitations. First, we do not have available data on glycated hemoglobin (HbA1c), which was previously reported to be a biomarker of diabetes and CVD damage, independently of fasting glucose [23]. Thus, new studies are needed to evaluate if lysine pathway metabolites may add value to the prediction of risk provided by HbA1c. Moreover, we did not find an effect of MedDiet on 1 year-changes in none of the studied metabolites. It may be possible that 1 year is not enough time to observe sound changes in metabolites, and as a consequence we did neither observe an association between 1-year changes of metabolites and the subsequent risk of T2D or CVD. Another limitation is that our results may be not generalizable because we used a Mediterranean population at high CVD risk. In addition, we cannot exclude the possibility of residual confounding regarding the observational associations between metabolites and cardiometabolic outcomes.

Consequently, these results, especially those suggesting a potential interaction between the nutritional intervention and these 2 metabolites deserve further confirmation in independent prospective cohort studies and trials [24]. However, our study presents important strengths: we used the case‐cohort design, which retains randomization, to both evaluate associations of 2-AAA and lysine with incident T2D and CVD. Our analyses were adjusted for multiple confounders within a well-characterized trial and, as an important novelty, we used repeated measurements of these metabolites, that allowed us to assess 2-AAA and lysine changes due to the dietary intervention.

## Conclusions

In conclusion, our results provide an interesting independent replication of the association of 2-AAA with future risk of T2D, a similar association for lysine and pipecolic acid and a suggestion of a diabetes-dependent association of lysine with subsequent CVD risk.

## Supplementary information

**Additional file 1.** Additional figures and tables.

## Data Availability

The datasets generated and/or analyzed during the current study are not publicly available due to the lack of authorization from PREDIMED participants but are available from the corresponding author on reasonable request.

## Abbreviations

2-AAA: 2 aminoadipic acid
CVD: cardiovascular disease
EVOO: extra-virgin olive oil
HR: hazard ratio
LC–MS: liquid chromatography–tandem mass spectrometry
LTPA: leisure time physical activity
MedDiet: Mediterranean diet
PREDIMED: PREvención con DIeta MEDiterránea
SD: standard deviation
T2D: type 2 diabetes
