# Supplementary material for: Lysine pathway metabolites and the risk of type 2 diabetes and cardiovascular disease in the PREDIMED study: results from two case-cohort studies
Source: Cardiovasc Diabetol. 2019 Nov 13;18:151. doi: 10.1186/s12933-019-0958-2 (PMC6852717; doi:10.1186/s12933-019-0958-2)
Supplement: Supplementary file 1 — Additional file 1. Additional figures and tables. [file 12933_2019_958_MOESM1_ESM.dotx]

**Supplementary material**

Other degradation pathways

N-acetyl-lysine

6-acetamido-

2oxohexanoate

6-amino-

2oxohexanoate

**L-pipecolate**

Penicilins and cephalosporins biosynthesis

**L-lysine**

L-2-aminoadipate

6-semialdehide

Saccharopine
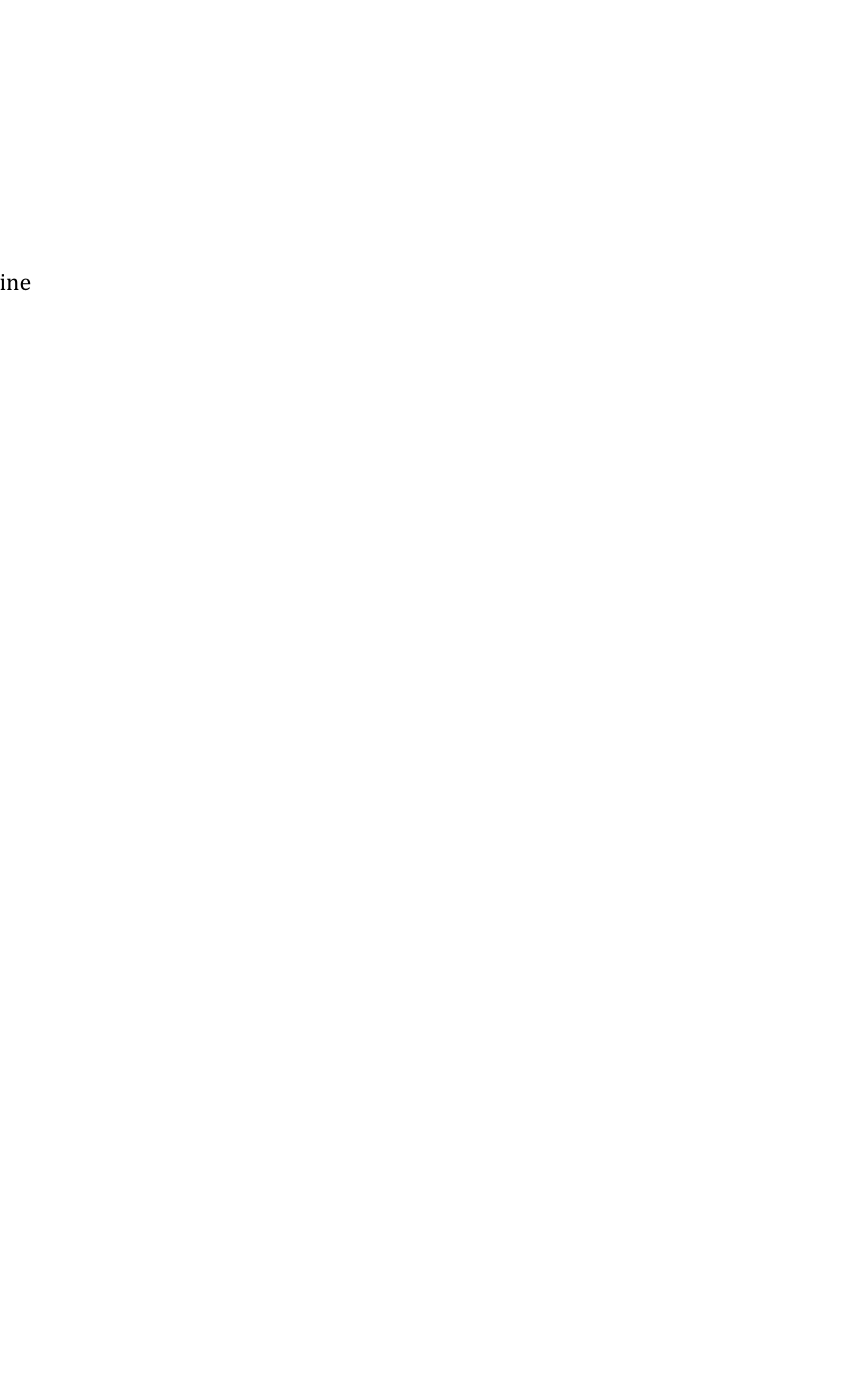
L-lysine

**L-2-aminoadipate**

alpha-aminoadipic semialdehyde synthase

aldehyde dehydrogenase

Lysine N-acetyltransferasa

N6-acetyl-beta-lysine transaminase

acyl-lysine deacylase

2-oxoadipate

2-oxoadipate

S-glutaril-dihidrolipoamide

Citrate cycle

kynurenine/2-aminoadipate aminotransferase

2-oxoglutarate dehydrogenase

dihydrolipoamide S-succinyltransferase

1-piperideine-2-carboxylate

Δ1-piperideine-

2-carboxylate

**Figure S1**. Lysine degradation pathway involving 2- aminoadipic acid and pipecolic acid.

Modified from www.kegg.jp


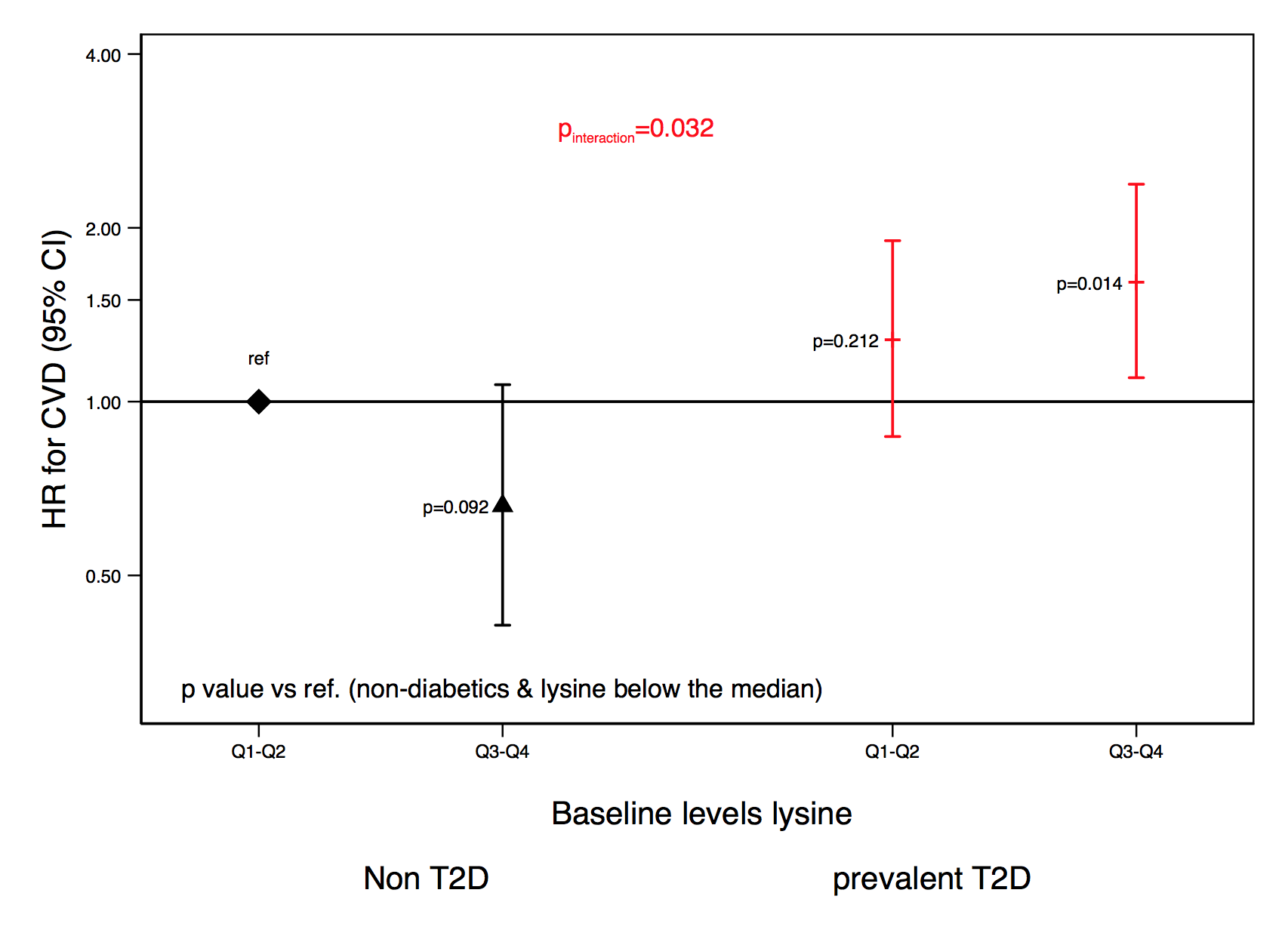


**Figure S2**. Multivariate adjusted hazard ratios (HRs; 95% confidence intervals [CIs]) of incident cardiovascular disease for baseline lysine values (above/below median) stratified by prevalent type 2 diabetes at baseline. P values are referred to the reference category composed of non-diabetic subjects with metabolite levels below the median.

The p value for the comparison of diabetics with lysine above the median vs diabetics with lysine below the median was 0.111.


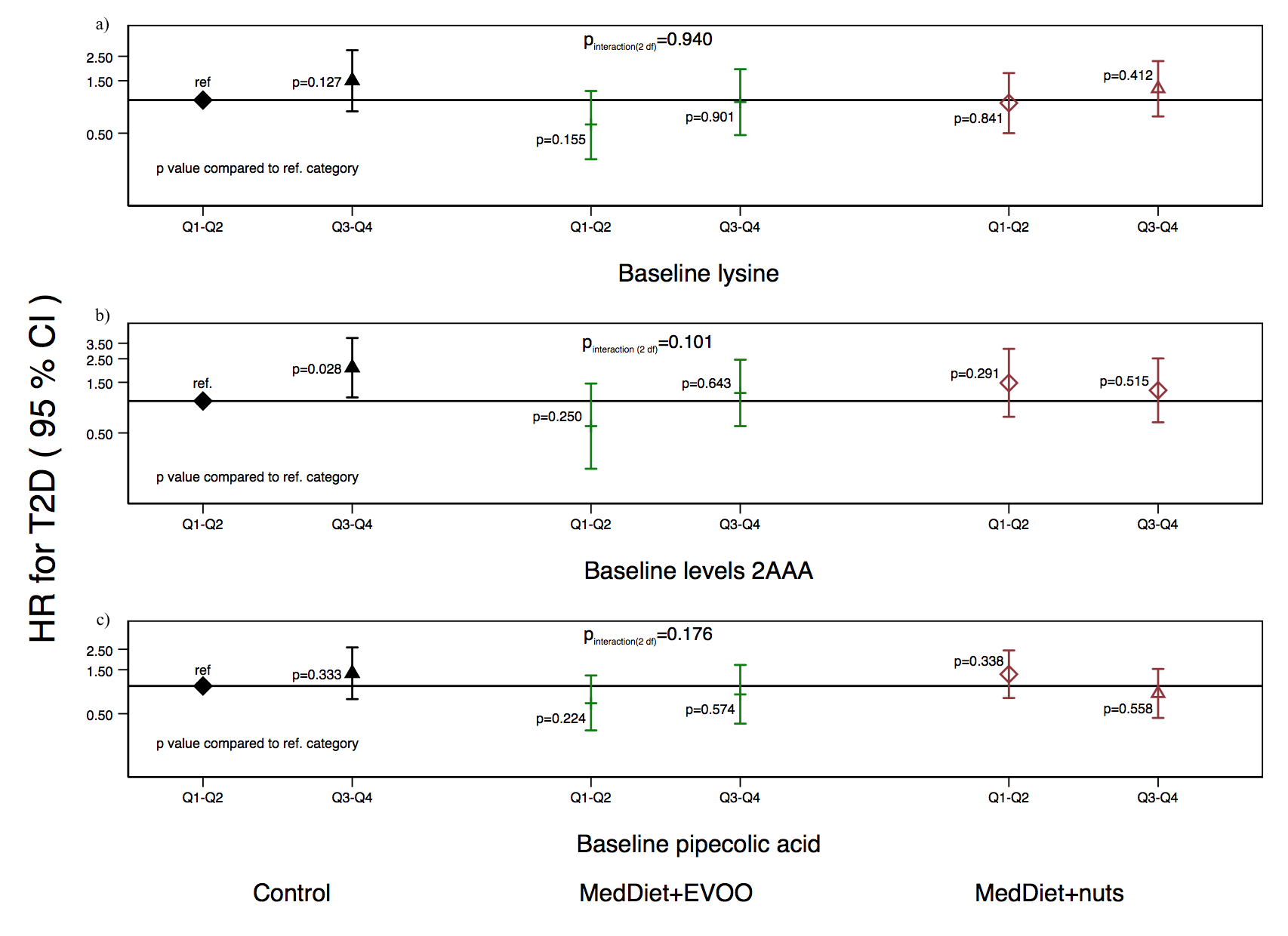


**Figure S3**. Multivariate adjusted hazard ratios (HRs; 95% confidence intervals [CIs]) of incident type 2 diabetes for quartiles (Q) of baseline a) lysine, b) 2-AAA and c) pipecolic acid stratified by intervention group.

P values are referred to the reference category composed of control group subjects with each baseline metabolite below the median.


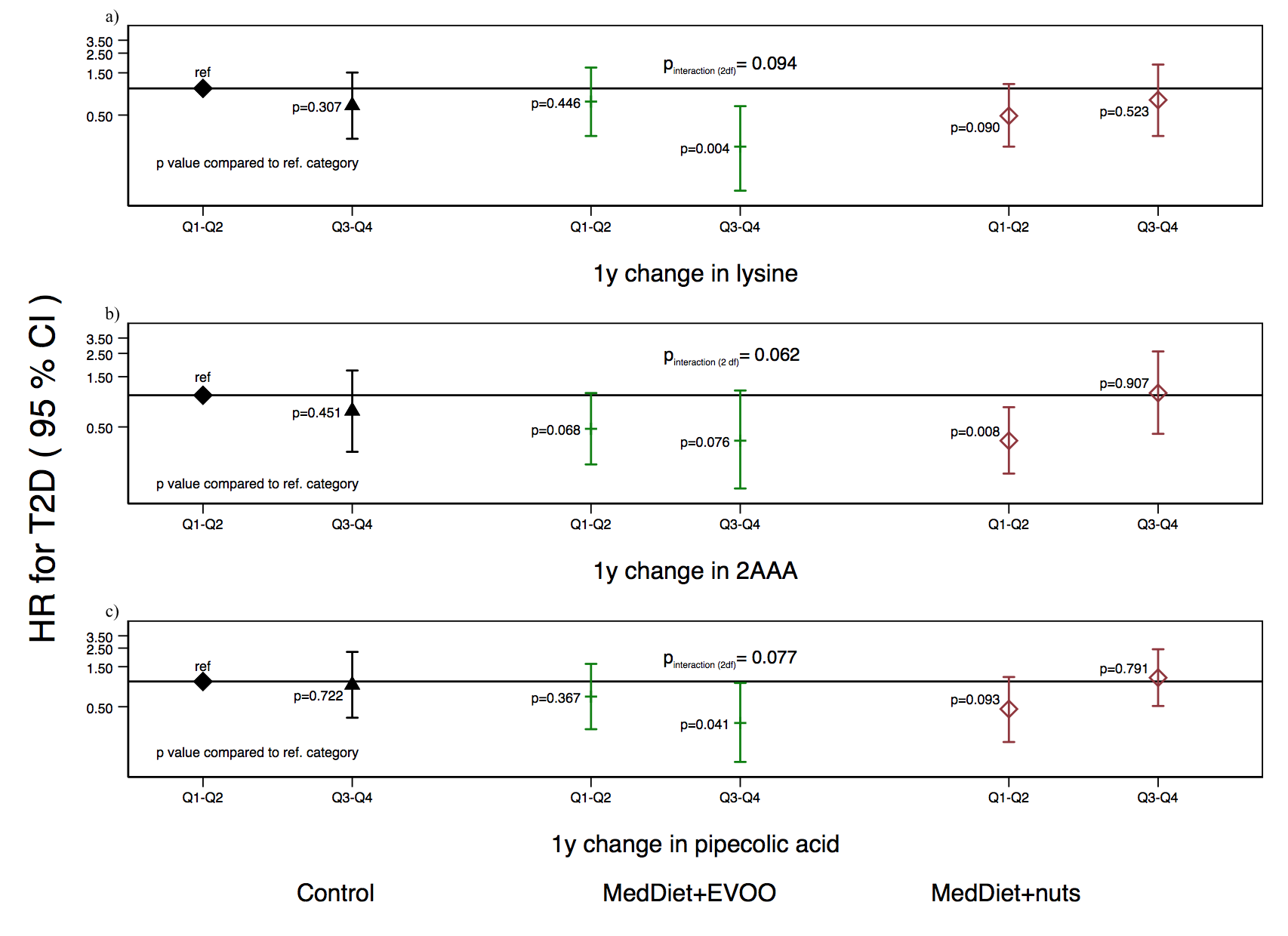


**Figure S4**. Multivariate adjusted hazard ratios (HRs; 95% confidence intervals [CIs]) of incident type 2 diabetes for quartiles (Q) of 1-y changes in a) lysine, b) 2-AAA and c) pipecolic acid stratified by intervention group.

P values are referred to the reference category composed of control group subjects with each baseline metabolite below the median.


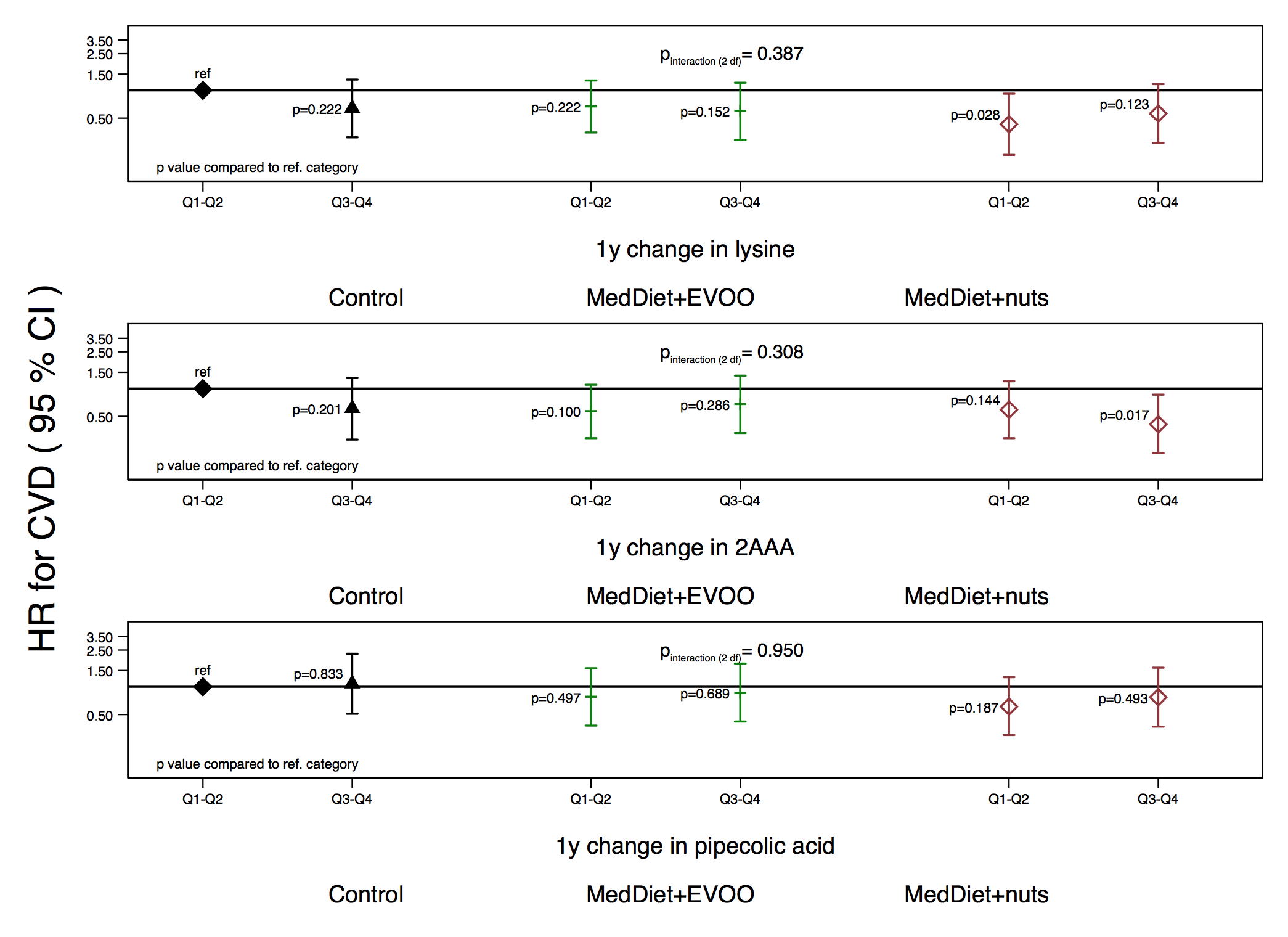


**Figure S5**. Multivariate adjusted hazard ratios (HRs; 95% confidence intervals [CIs]) of incident cardiovascular disease for quartiles (Q) of 1-y changes in a) lysine, b) 2-AAA and c) pipecolic acid stratified by intervention group.

P values are referred to the reference category composed of control group subjects with each baseline metabolite below the median.

|  | **MedDiet+EVOO vs control** | | **MedDiet+Nuts vs control** | |
| --- | --- | --- | --- | --- |
|  | **β (95%CI)** | **p** | **β (95%CI)** | **p** |
| **2-AAA 1-y change*** | 0.031 (-0.077 to 0.138) | 0.575 | -0.005 (-0.116 to 0.104) | 0.921 |
| **Lysine 1-y change*** | 0.043 (-0.058 to 0.145) | 0.402 | 0.001 (-0.103 to 0.106) | 0.977 |

**Table S1.** Effect of 1-y intervention on 1-y changes of 2-AAA and lysine adjusted for age, sex. BMI, family history of CVD, smoking, leisure-time physical activity, education level, type 2 diabetes, hypertension and dyslipidaemia.

*Inverse normally transformed residual change.
